# Semantic Segmentation and Depth Estimation Based on Residual Attention Mechanism

**DOI:** 10.3390/s23177466

**Published:** 2023-08-28

**Authors:** Naihua Ji, Huiqian Dong, Fanyun Meng, Liping Pang

**Affiliations:** 1 School of Information and Control Engineering, Qingdao University of Technology, Qingdao 266033, China; 13964863452@126.com (N.J.); 18235278465@163.com (H.D.); 2 School of Mathematical Sciences, Dalian University of Technology, Dalian 116024, China; lppang@dlut.edu.cn

**Keywords:** Semantic segmentation, depth estimation, residual attention, gradient balance

## Abstract

Semantic segmentation and depth estimation are crucial components in the field of autonomous driving for scene understanding. Jointly learning these tasks can lead to a better understanding of scenarios. However, using task-specific networks to extract global features from task-shared networks can be inadequate. To address this issue, we propose a multi-task residual attention network (MTRAN) that consists of a global shared network and two attention networks dedicated to semantic segmentation and depth estimation. The convolutional block attention module is used to highlight the global feature map, and residual connections are added to prevent network degradation problems. To ensure manageable task loss and prevent specific tasks from dominating the training process, we introduce a random-weighted strategy into the impartial multi-task learning method. We conduct experiments to demonstrate the effectiveness of the proposed method.

## 1. Introduction

Computer vision has many important underlying disciplines, such as visual target tracking [1], scene understanding, and so on. Scene understanding is a crucial problem in computer vision that encompasses various aspects, such as semantic labeling to identify different parts of the scene, depth estimation [2] to describe the physical geometry, and instance segmentation [3]. Two fundamental tasks in scene understanding are depth estimation and semantic segmentation, which have been extensively studied using deep learning. Previous networks were designed to perform only one of these specific tasks [4,5]. However, recent works [6,7,8] have found interactions between these two tasks and achieved significant performance gains by exploiting their common features to promote each other. In the field of computer vision systems, it is generally preferable to perform multiple tasks concurrently rather than focusing on just one. This approach enables the system to learn both tasks simultaneously, which is more efficient and effective.

Given the advantages of multi-task learning, the goal of our research is to perform joint learning of semantic segmentation and depth estimation. When considering multi-task learning, it is important to focus on two main aspects: the structure of the network model used for prediction and the balance between tasks. Although many methods have been proposed for joint learning of two tasks, previous studies have tended to consider only one of these aspects. Also, in terms of task balancing, previous studies have only considered one of either gradient balancing or loss balancing, without taking both considerations into account. A successful network framework for multi-task learning must represent both shared features of individual tasks (to prevent overfitting) and task-specific features (to prevent underfitting). There is a popular approach to deal with multi-task learning: the multi-task attention network (MTAN) [9]. The MTAN facilitates the learning of task-specific features at the feature level, and it enables the learning of task-specific features from global features, while also allowing for feature-sharing across multiple tasks. However, this model structure has too many parameters and requires considerable time to train. In multi-task learning, it is crucial to maintain a balance between the learning of different tasks, as some may be better learned than others due to varying loss sizes or gradient sizes. To tackle this challenge, a number of approaches have been put forth, including gradient adjustment techniques, such as gradient magnitude normalization [10] and Pareto optimality [11], as well as loss adjustment techniques such as homoskedasticity uncertainty [12]. The literature [13] has considered both gradient adjustment and loss adjustment. Although the method provided in [13] can keep the task loss on a controllable scale, it is easily dominated by some specific tasks. 

**Figure 1:**
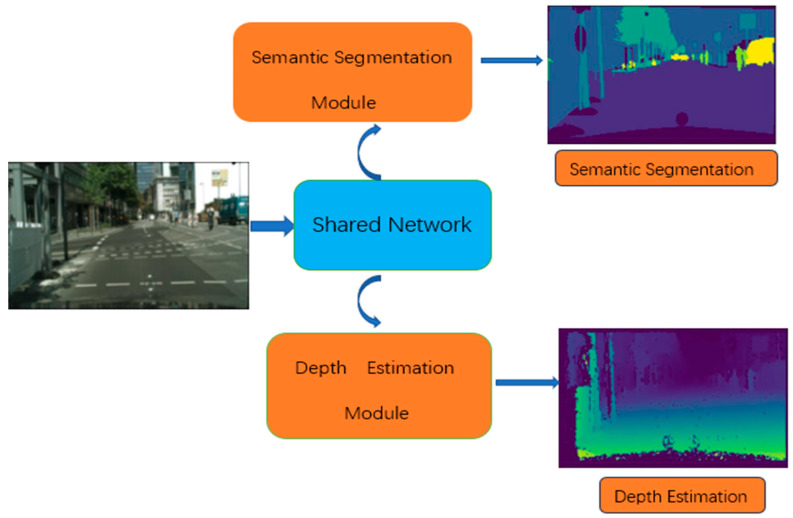
Illustration of the MTRAN.

To address the above problems, we propose a multi-task residual attention network (MTRAN). The MTRAN mainly consists of a shared network and two task-specific networks, as shown in Figure 1; the shared network extracts global features from the input image, and two task-specific modules handle semantic segmentation and depth estimation. The network introduces a residual attention module; the attention module enables the network to focus on the important information in the image, on top of which residual connections are added, with the aim of solving the network degradation problem that occurs as the number of layers of the network deepens, while also improving the training speed and accuracy. Furthermore, we use a convolutional block attention module (CBAM) to emphasize the global feature map through additive operations, which makes up for the shortcomings of the MTAN. From the optimization method aspect, to maintain task losses on a more balanced and controllable scale, we combine the random-weighted strategy and impartial multi-task learning together by dynamically adjusting the weightings, and the gradients are also balanced.

The main contributions of this paper can be summarized as follows.

- The article proposes a multi-task residual attention network, which introduces a residual attention module to perform end-to-end multi-task learning. The MTAN [9] uses a soft attention mask to extract features of interest from shared features, and we also use an additive operation to emphasize global features before extracting features of interest using the CBAM, and the residual connectivity is added to improve the training speed and accuracy.
- To keep the loss of tasks on a controllable scale and prevent the training process from being dominated by specific tasks, a random-weighted strategy is added to the IMTL method. In this way, the losses and gradients are further balanced impartially and dynamically.
- The experiments are conducted to demonstrate the effectiveness of the proposed method. Meanwhile, some comparison experiments are carried out with other related methods.

## 2. Related Work

### 2.1. Multi-Task Learning

Humans are capable of learning multiple related tasks simultaneously and can leverage knowledge from one task to improve learning in another. Similarly, multi-task learning in deep learning involves training on multiple related tasks to utilize the information contained in each task to enhance learning. In real-world applications, multi-task learning is more efficient than working on one task at a time, and the results can be improved due to the use of information from other tasks.

Multi-task learning (MTL) has been extensively applied in diverse fields of machine learning, including computer vision [14,15], sentence representation learning [16], sentiment analysis [17], action recognition [18], and instance perception [19]. The architecture of the network for multi-task learning is a crucial obstacle, requiring a balance between a competent shared feature representation and a task-specific feature representation. Additionally, MTL networks must maintain the ability to learn a general feature representation of the data while learning a feature representation for each task to avoid overfitting.

Xie et al. utilized a multi-task attention-guided network to handle multi-objective fault diagnosis with small samples [20]. It uses the same dynamic weighted average as the MTAN in terms of task-balancing considerations. Zhang et al. proposed a new framework called task-recursive learning (TRL) for joint semantic segmentation and depth estimation [21]. The TRL framework serializes the problem as a task-alternating time series, which progressively improves and mutually promotes both tasks by appropriately propagating the information flow. Gao et al. proposed a context-informed network (CI-Net) for multi-task learning of semantic segmentation and depth estimation [22]. Both of these studies simply weighted the losses linearly. In contrast, our approach not only goes for dynamic balancing of task loss but also considers it in terms of gradient balancing.

### 2.2. Residual Attention

In the field of computer vision, attention has been utilized as a crucial technique to enhance the performance of CNNs in various tasks, including classification and target detection. The objective of attention is to concentrate on task-relevant data to improve accuracy. Attention mechanisms are generally categorized into two types: spatial domain attention and channel domain attention. Many works have studied these two types of attention, such as [23,24,25,26,27]. Based on channel domain attention, Hu et al. proposed a squeeze-and-excitation (SE) module [23], which aims to learn the inter-channel relations of different convolutional channels. Furthermore, Wang et al. utilized an efficient channel attention (ECA) module [24] to mitigate the issue of dimensionality reduction caused by the SE module and effectively obtain cross-channel interaction information. Woo et al. proposed a convolutional block attention module (CBAM) by combining the spatial and channel domains [25]. Zhang et al. utilized the self-attention mechanism to capture robust contextual information to improve the feature representation of the network [26]. Fu et al. introduced a self-attention mechanism to capture feature dependencies in spatial and channel dimensions, respectively [27].

The performance of a neural network can be improved by increasing its width and depth, but simply increasing the depth may cause the gradient to disappear or explode during backpropagation using the chain rule, leading to network degradation. To address this issue, Kai-Ming He et al. proposed a solution in the form of a deep residual network [28], which effectively prevents network degradation; this explicitly reformulates the layers as learning residual functions with reference to the layer inputs, instead of learning unreferenced functions. Chen et al. use the idea of residuals for joint learning of facial landmark localization and expression recognition [29]. Devvi Sarwinda et al. investigated a deep-learning image classification method based on ResNet architecture for colorectal cancer detection [30]. While all of these networks use the idea of residuals to enhance the network, they lack the attention mechanism to focus on task-specific features, in contrast to our approach, which combines the idea of residuals with the attention mechanism.

## 3. Methods

In this section, we propose the MTRAN model. It contains a shared network and two task-specific networks, both based on the SegNet network as the backbone. The encoder part of the task-specific network consists of a convolutional module, and the decoder part consists of an attentional module and a convolutional module. An overview of the MTRAN structure is given in Section 3.1, after which we present the specific structures of the shared network and the task-specific networks in Section 3.2 and Section 3.3, respectively. In addition, for the loss function optimization, we introduce random-weighted loss and impartial multi-task learning, which will be introduced in Section 3.4. 

### 3.1. Network Architecture

**Figure 2:**
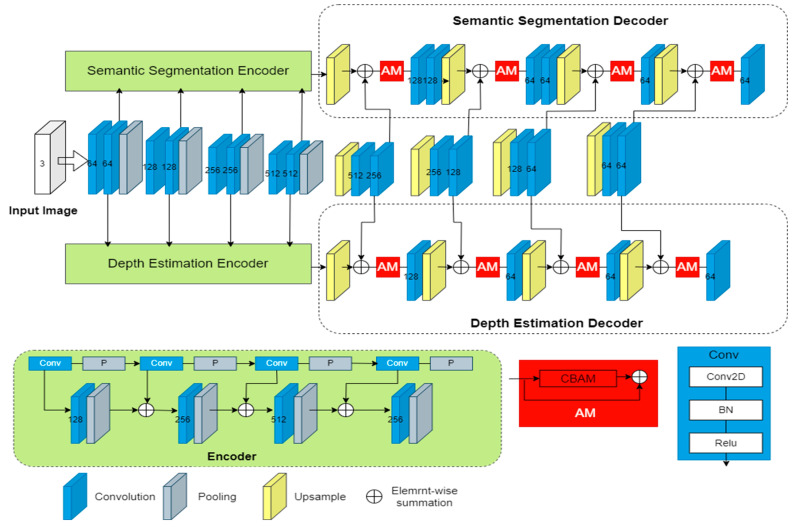
The network framework of the MTRAN.

The whole network architecture consists of two components: a shared network and two task-specific networks. The shared network is responsible for learning a set of global features that are applicable to all the tasks. On the other hand, the attention module in each task-specific network decoder section is linked to the shared network and serves as a feature selector, allowing the network to learn task-specific features. A visual representation of this architecture can be found in Figure 2.

Our approach utilizes SegNet as the underlying network, which employs an encoder–decoder architecture. The encoder module is responsible for extracting object information, whereas the decoder module maps the extracted information onto the image space. 

The shared network is mainly composed of convolutional modules, and we also cut the number of layers and convolutional modules of SegNet in order to speed up the training. The two task-specific networks are used to handle the semantic segmentation and depth estimation tasks, respectively, with the encoder part consisting of convolutional modules, and the decoder part consisting of a series of residual attention modules and convolutional modules. It is important to note that the first module just takes the shared features as input, while each subsequent module adds the output of the previous module to the shared features of this layer as input. As the findings in the literature [24] show, better performance can be achieved if the segmentation decoder is larger than the depth decoder. Therefore, in our network architecture, we designed the split-decoder network to be more complex than the depth-decoder network.

In Figure 2, the Conv module represents the convolution operation and consists of three parts: a 2D convolutional layer, a batch normalization layer, and a Relu activation function layer. The Attention Module (AM) will be described in detail in Section 3.3. The 2D convolutional layer is defined as follows:

(1) $${f\;}_{\mathrm{out}}\;=\sum_{i=0}^{k}\sum_{j=0}^{k}W\left(i,j\right)*X(k-i,k-j)$$

where *X* denotes the matrix of the input convolutional layer, *W* denotes the weight matrix, *i*, *j* is the position of a pixel point, and *k* denotes the size of the convolutional kernel.

The convolutional layer is followed by a batch normalization layer defined as follows:

(2) $$x_{i}^{\prime }=\frac{X_{i}-\mu }{\sqrt{\sigma^{2}}}$$

where μ and σ are the mean and variance, respectively, and are defined as

(3) $$\mu =\frac{1}{m}\sum_{i=1}^{m}x_{i}$$

(4) $$\sigma =\frac{1}{m}\sum_{i=1}^{m}{\left(x_{i}-\mu \right)}^{2}$$

where *m* denotes the mini-batch size. This is followed by a Relu function activation layer that aims to keep the better values of the features, and round off the values with features less than 0; the Relu function is defined as follows:

f(*x*) = max(0, *x*) (5)

In addition, pooling and upsampling in Figure 2 denote the pooling and upsampling layers, and our model uses maximum pooling, which means that the maximum value in the sliding window is selected as the pooled value of the region, the upsampling will restore the maximum value to the corresponding position, and then the other positions are complemented with 0.

### 3.2. Task-Sharing Network

The shared network is based on SegNet; to improve the training speed, we modified SegNet. The encoder and decoder parts of the modified network are both only four layers. The encoder has two convolution modules and pooling operations for each layer. The decoder part has upsampling operations and two convolution modules for each layer, and the convolution module consists of the convolution part, normalization processing, and Relu function activation. The size of the convolution kernels used in the convolution module are all 3 × 3. The number of channels in the encoder part is 64, 128, 256, and 512, respectively. The number of channels in the decoder part is slightly different, and the number of channels in the convolution module in each layer is 512 and 256, 256 and 128, 128 and 64, 64 and 64, respectively.

### 3.3. Task-Specific Networks with Residual Attention

(1) Semantic segmentation network

The semantic segmentation network contains encoder and decoder parts; the encoder has one convolutional module and pooling operation in each layer, and the number of channels is 128, 256, 512, and 256, respectively. Each layer of the decoder contains the upsampling operation, attention module, and convolutional module. The details are shown in Figure 2, where the first and second layers of the decoder part have two convolutional modules, and the remaining two layers have one convolutional module. The number of channels is 128, 64, 64, 64, respectively.

(2) Depth estimation network

The encoder section of the depth estimation network is identical to the encoder section of the semantic segmentation network. Additionally, the number of channels in the decoder section of both networks is the same. However, the decoder section of the depth estimation network consists of convolutional modules for each layer, whereas the first and second layers of the semantic segmentation network’s decoder section contain two convolutional modules. This is because previous research [31] has shown that a larger segmentation decoder improves performance compared to the depth decoder.

The paper proposes a novel approach for attention-based networks that utilizes CBAM. Unlike the MTAN, which uses a soft attention mask to eliminate irrelevant features, the proposed approach uses the CBAM additivity operation to emphasize the global feature map. The attention mechanism is divided into spatial and channel attention, and CBAM combines both through its two submodules, the channel attention module (CAM) and the spatial attention module (SAM). CBAM can be easily integrated into existing network structures as a plug-and-play module. The proposed approach also incorporates residuals inspired by [28], which improves the network’s speed and accuracy. The authors found that the best performance is achieved when the input first goes through CAM before SAM.

Given K tasks: T = {$t_{1}\ldots t_{i}\ldots t_{K}$}, i denotes the index of each task, and for each task i, we use *j* to denote the index of each layer. The shared features of the *j*th layer in the shared network are denoted by $S_{j}$; the features input to the convolution module in the encoder part are denoted by $c_{i}^{j}$, and the features learned by the convolution module are denoted by ${\hat{c}}_{i}^{j}$; the features input to the attention module in the decoder part are denoted by $a_{i}^{j}$, and the features learned by the attention and convolution modules are denoted by ${\hat{a}}_{i}^{j}$. The formulas of $c_{i}^{j}$ and $a_{i}^{j}$ are given as follows:

(6) $$\left\{\begin{array}{c} c_{i}^{j}=S_{j}\;\;\;\;\;\;\;\;\;\;\;\;\;\;\;\;\;\;\;\;\;j=1\\ c_{i}^{j}=S_{j}⊕{\hat{c}}_{i}^{j-1}\;\;\;\;2\leq \;j\leq 4 \end{array}\right.$$

(7) $$\left\{\begin{array}{c} a_{i}^{j}=S_{j}⊕{\hat{c}}_{i}^{j-1}\;\;\;\;\;\;\;\;\;\;\;\;\;\;\;j=5\\ a_{i}^{j}=S_{j}⊕{\hat{a}}_{i}^{j-1}\;\;\;\;\;\;\;\;\;\;\;\;\;\;\;j\geq 6 \end{array}\right.$$

The first attention module for each task takes the shared features as input. Each subsequent piece is an additive operation between the shared features and the output of the previous layer. ${\hat{c}}_{i}^{j}$ and ${\hat{a}}_{i}^{j}$ are defined as follows:

(8) $${\hat{c}}_{i}^{j}=\mathrm{Conv}(c_{i}^{j})$$

(9) $${\hat{a}}_{i}^{j}=\;\mathrm{Conv}(\mathrm{SAM}(\mathrm{CAM}(a_{i}^{j}))\;⊕a_{i}^{j})$$

The operations of the CAM and SAM are defined as follows. Firstly, in the CAM, the $a_{i}^{j}$ is respectively maximum pooled and global average pooled to obtain two 1 × 1 × *c* (*c* denotes the number of channels) feature maps. Then, they are respectively fed into a two-layer neural network, and the output features are summed and operated on; after activation with the sigmoid function, the final channel attention feature is obtained, and finally, the feature is multiplied with the input feature $a_{i}^{j}$ to generate the SAM-required features. The input features to the SAM are subjected to channel-based global maximum pooling and global average pooling to obtain two feature maps and splice them together; then, they are downscaled by a convolution operation to a feature map of one channel, and then they are subjected to a sigmoid function to generate a spatial attention feature, which is then multiplied with the input spatial attention feature to generate the final features after the CBAM. Finally, we introduce residual concatenation, which is the summing operation of the final feature after the CBAM with the original input $a_{i}^{j}$.

### 3.4. Model Training

In this section, we define the loss function of the model and the optimization algorithm. For semantic segmentation, we use the cross-entropy loss:

(10) $$L_{seg}=-\sum_{i=1}^{N}y_{i}log{\hat{y}}_{i},$$

For depth estimation, we use the absolute error:

(11) $$L_{dep}=\sum_{i=1}^{N}\left|y_{i}-{\hat{y}}_{i}\right|,$$

where $y_{i}$ denotes the label value, and ${\hat{y}}_{i}$ denotes the model output value. We define the total loss function as:

(12) $$L_{total}=w_{1}L_{seg}+{w_{2}L}_{dep},$$

where $w_{1}\;and\;w_{2}$ are the weights for the semantic segmentation and depth estimation, respectively.

To prevent the training process from being dominated by one task, we use the random-weighted strategy, which means that the weights obey the Dirichlet distribution. The probability density function obeyed by the Dirichlet distribution is defined as follows:

(13) $$p(P,\alpha )=\frac{1}{B(\alpha )}\prod_{i=1}^{k}p_{i}^{\alpha_{i}-1},$$

where *k* ≥2, K denotes the number of tasks, P = [$p_{1},\ldots ,p_{n}$], $\sum_{1}^{K}p_{1}$ = 1, $p_{i}$ is the weight of the *i*th task, *B*(α) is the normalization constant, and α = [$\alpha_{1},\ldots ,\alpha_{K}$], which can be expressed as a gamma function:

(14) $$B(\alpha )=\frac{\prod_{i=1}^{k}\Gamma (\alpha_{i})}{\Gamma (\sum_{i=1}^{K}\alpha_{i})},$$

To keep the loss at a controllable scale and make the gradient in the balance, we use the IMTL [13] optimization method to train the network. IMTL uses the balance losses and gradient balances together. In IMTL-loss, the multi-task losses are controlled by scale parameters, which are continuously updated during the training process and reduce the training speed. The Dirichlet distribution is a high-dimensional continuous distribution and is suitable for multiple tasks. To increase the computing speed, we replace the balance losses with random-weighted losses, which satisfies the Dirichlet distribution. The random-weighted loss function can prevent the learning procedure from being dominated by any specific tasks. In each training batch, two numbers are generated that obey the Dirichlet distribution and have a sum of 1. The two numbers are between 0 and 1. Then, these two numbers are used as weights for each task to weigh and calculate the total loss, after which the gradient is calculated using the total loss. Finally, the gradient is adjusted using IMTL-Gradient with the following adjustment strategy. The main objective of IMTL-Gradient is that the projection of aggregated gradient onto each task gradient is equal. Assume

(15) $$g=\sum_{t=1}^{T}\beta_{t}g_{t},\;\mathrm{where}\;g_{t}=\nabla L_{total}\left(\theta \right)$$

and let $u_{t}$ = $\frac{g_{t}}{\left||g_{t}|\right|}$, then, we obtain that

(16) $$gu^{T}={gu}_{t}^{T},\;\mathrm{which}\;\mathrm{is}\;g(u^{T}{-u}_{t}^{T})=0,\;2\leq t\leq T$$

Assuming the weight of gradients satisfies $\sum_{t=1}^{T}\beta_{t}=1$, denote β = [$\beta_{2},\ldots ,\beta_{T}$], $U^{T}$ = [$u_{1}^{T}-u_{2}^{T},\ldots ,u_{1}^{T}-u_{T}^{T}$], and $G^{T}$ = [$g_{1}^{T}-g_{2}^{T},\ldots ,g_{1}^{T}-g_{T}^{T}$], by a simple computation on (16), we obtain that β = $g_{1}U^{T}{\left(DU^{T}\right)}^{-1}$(IMTL-G).

Then, the weight of the gradients is $\tilde{\beta }$ = (1−Iβ,β), where I = (1, …, 1), and β∈IMTL-G.

The IMTL-Gradient (IMTL-G for short) algorithm with random-weighted multi-task losses is given as follows (Algorithm 1).
**Algorithm 1** IMTL-G algorithm with random-weighted multi-task losses**Input**: Initialized task-shared/specific parameters $\theta_{sh}$/$\left\{\theta_{t}\right\}$ and learning rate η **Output**: 1. **for** *t* = 1 to *T* **do** 2.   compute task scaled loss: $L_{1}$,…$L_{t}$ 3.   compute weight: $w_{t}$~Dirichlet 4.   compute total loss: $L_{total}$ = $\sum_{t=1}^{T}w_{t}L_{t}$ 5.   compute gradient of shared feature: $g_{t}$ = $\nabla L_{total}$ 6.   compute unit-norm gradient $u_{t}$ = $\frac{g_{t}}{\left||g_{t}|\right|}$ 7. **end for** 8. $\tilde{\beta }$ = (1−Iβ,β), where I = (1,…,1), β∈ IMTL-G 9. update task-shared parameters $\theta_{sh}$ = $\theta_{sh}$ − $\eta \nabla_{\;\theta_{sh}}$($\sum_{t=1}^{T}{\tilde{\beta }}_{t}L_{t}$) 10. **for** t = 1 to T **do** 11.   update task-specific parameters $\theta_{t}$ = $\theta_{t}-$ $\eta \nabla_{\;\theta }L_{t}$ 12. **end for**

## 4. Experiment

In this section, the proposed MTRAN is evaluated based on three simulation cases. Firstly, we present the ablation studies, then, the visualization results are provided, and finally, the proposed method is compared with other methods.

### 4.1. Dataset

CityScapes: The CityScapes [32] dataset is mainly used to evaluate the performance of vision algorithms for semantic understanding of urban scenes, and it contains street scenes of 50 cities with different scenes and seasons, with 5000 frames of high-quality pixel-level annotations and 20,000 frames of weak annotations. We set all the training and test images to a size of 128 × 256. The dataset contains 2, 7, and 19 classes of label groupings, and we use 7 classes of labels for multi-task learning of semantic segmentation and depth estimation.

NYUv2 dataset: The NYUv2 dataset [33] consists of RGB-D indoor scene images. We evaluate performance on two learning tasks: 13-class semantic segmentation and depth estimation. We processed the dataset in the same way as the MTAN [9], resizing all the training and test images to a resolution of 288 × 384.

### 4.2. Training Setup

The experiments were all implemented in Pytorch. The batch size is set to 8, the initial learning rate is set to 0.001, and the model is trained for 200 epochs. For the semantic segmentation task, we use mIou and pixel accuracy as metrics, and the definitions are as follows:

(17) $$\mathrm{mIou}=\frac{1}{k+1}\sum_{i=0}^{k}\frac{p_{ii}}{\sum_{j=0}^{k}p_{ij}+\sum_{j=0}^{k}p_{ji}-p_{ii}}$$

(18) $$\mathrm{Pix}\;\mathrm{Acc}=\frac{\sum_{i=0}^{k}p_{ii}}{\sum_{i=0}^{k}\sum_{j=0}^{k}p_{ij}}$$

*k* denotes the category, *i* denotes the true value, *j* denotes the predicted value, and $p_{ij}$ denotes the prediction of *i* as *j*.

And, for the depth estimation task, the absolute and relative errors are used to evaluate the performance; the definitions are as follows:

(19) $$\mathrm{Abs}\;\mathrm{Err}=\sum_{i=1}^{N}\left|y_{i}-{\hat{y}}_{i}\right|$$

(20) $$\mathrm{Rel}\;\mathrm{Err}=\sum_{i=1}^{N}\frac{\left|{\hat{y}}_{i}-y_{i}\right|}{y_{i}}$$

$y_{i}$ denotes the label value, and ${\hat{y}}_{i}$ denotes the model output value.

### 4.3. Ablation Studies

The ablation studies are conducted to demonstrate the effectiveness of each component of the proposed MTRAN network structure. Keeping the backbone network and task-specific convolutional module components unchanged, we compare the results of experiments with and without residual CBAM, and with the addition and removal of RWL, respectively.

(1) Effectiveness of residual attention

The ablation experiments are used to verify the effectiveness of residual attention. The comparison results are shown in Table 1. From Table 1, we can see that both semantic segmentation and depth estimation have better results after adding residual CBAM, which indicates that the attention module helps to improve the accuracy of multiple tasks. The MTRAN denotes our model, and without-att denotes the removal of the residual attention module.

**Figure 3:**
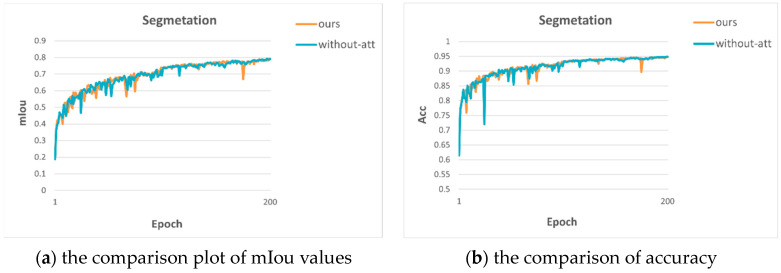

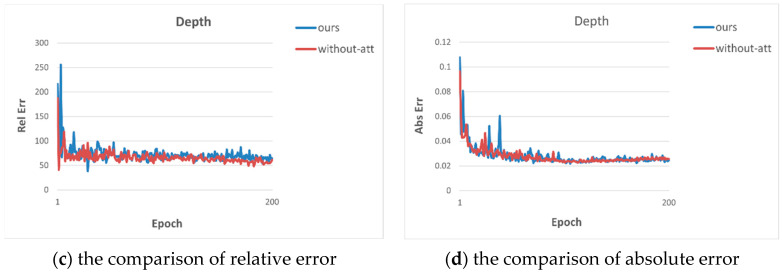
Comparison plots for two structures.

We also show the comparison plots of metrics on semantic segmentation and depth estimation in Figure 3 to visualize the advantages of adding residual attention.

In order to verify the validity of the residual CBAM, we also conducted corresponding experiments on the NYUv2 dataset, and the results are shown in Table 2 and Table 3. Table 2 shows the comparison between our framework, removing attention and retaining attention. From Table 2, it can be seen that the addition of the attention mechanism gives the best results for both tasks.

Table 3 shows the comparison between our framework and the MTAN under neither optimization strategy. The MTAN uses a soft attention mask to extract features of interest from shared features, and we also use an additive operation to emphasize global features before extracting features of interest using residual CBAM. From Table 3, we can see that our network performs better than the MTAN on both tasks.

We also demonstrate the effectiveness of incorporating residual connectivity on the CityScapes dataset. The time to train an epoch is reduced from 14s to 13s after adding the residual connection, which improves the training speed by about seven percent, and the experimental results are shown in Table 4.

(2) Ablation experiments of optimization algorithms

In order to verify the benefits of RWL, we also conducted the corresponding ablation experiments on the CityScapes dataset, and the experimental results are shown in Table 5. From the table, we can see that after removing RWL, although the semantic segmentation effect is a little better, the depth estimation effect decreases considerably, proving that the addition of RWL plays a balancing role and prevents the multi-task learning from being dominated by a specific task.

**Figure 4:**
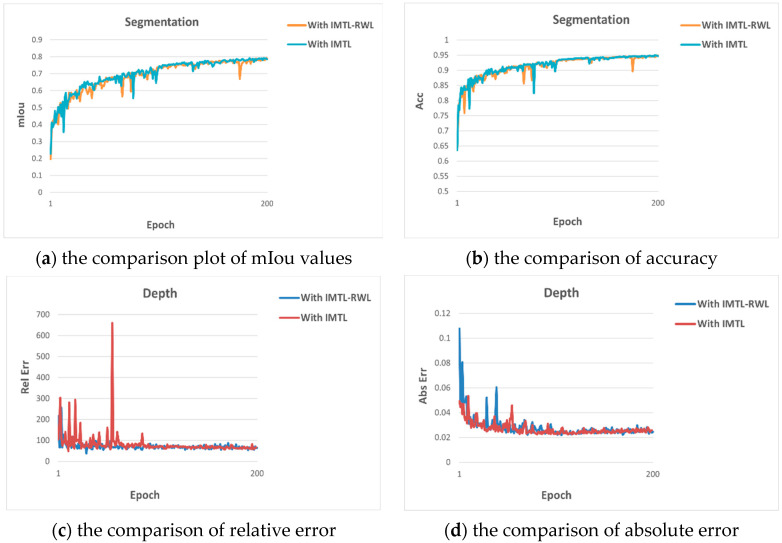
Comparison plots for two methods.

We also show the comparison plots of metrics on semantic segmentation and depth estimation in Figure 4 to visualize the advantages of adding RWL.

### 4.4. Visualization Results

**Figure 5:**
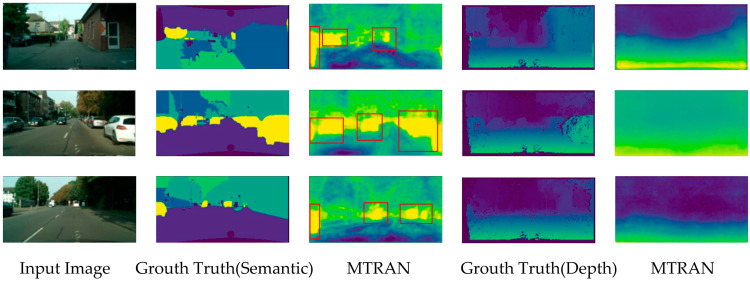
Visualization on CityScapes dataset.

To visually demonstrate the effectiveness of the proposed method, we visualize the model output results, and the result is shown in Figure 5. The first column in the graph is the original image, the second and fourth columns are the labels for the semantic segmentation and depth estimation, respectively, and the third and fifth columns are the output visualizations of our model.

This shows that the proposed model can achieve a better segmentation effect, especially for the cars. Our method can identify every vehicle in the image, even if it is the rear part of the vehicle in the alley or the part of the vehicle at the edge of the image; we have labeled these in Figure 5 with red boxes. But the sky is not well divided out, which is a direction for further improvement.

### 4.5. Comparison Results

We evaluate the performance of MTRAN using a 7-class CityScapes dataset, and we compare the network structure and task balance separately to confirm the effectiveness of the proposed approach.

#### 4.5.1. Comparison of Network Structure

First, we compare the proposed model with other methods, and the results are shown in Table 6, where #P denotes the number of network parameters, ↑ indicates a larger value is better, and ↓ indicates a smaller value is better. The best evaluation metrics are highlighted in bold.

Single-Task: Only one task and no attention.

Muti-Task Attention Network (MTAN): The multi-tasking attention network proposed by Liu [9] et al.

Deformable-Attention Multi-Task Network (DAMTN): Multi-task learning network based on geometric invariant discriminative features proposed by Liu Y [34] et al.

Multi-Task: Standard multi-task learning with prediction for each specific task only at the last layer, and no attention mechanism added. In addition, we add the proposed IMTL-RWL algorithm.

Cross-Stitch: Cross-stitch network [35] proposed by Misra I et al. An adaptive multi-task learning approach.

MTRAN (Ours): A multi-tasking structure with residual attention and impartial multi-tasking learning.

In Table 6, we can see that the number of parameters in our model is much fewer than the classical MTAN [9] method, and, although our model is not optimal in the depth estimation task, our method has the best performance in the semantic segmentation.

#### 4.5.2. Comparison between Task Balance

In this subsection, the optimization strategies in terms of task balancing are compared. The optimization method includes the proposed IMTL-RWL, multiple-gradient descent algorithm [11], conflict-averse gradient method [36], projecting conflicting gradients [37], and GradDrop [38]. The network structure is based on the MTRAN, and the results are shown in Table 7.

As can be seen in Table 7, although the GradDrop optimization method is optimal in terms of semantic segmentation, the depth estimation becomes worse. Although our method ranks second in semantic segmentation, the depth estimation effect is optimal and achieves a balanced performance.

## 5. Conclusions

In this work, we propose an MTRAN model for handling semantic segmentation and depth estimation multi-tasking, which can, of course, be used in other visual scene understanding multi-tasks, as well. The model is based on SegNet with the addition of residual CBAM, which combines attention in the channel and spatial domains to improve performance. We use random-weighted loss to determine task weights to prevent domination by specific tasks, then use the impartial multi-task learning method to balance the training processing. Although our method achieves good results in semantic segmentation and depth estimation multi-task learning, especially in semantic segmentation, there are some shortcomings, such as the training time being too long, as well as the depth estimation effect being sub-optimal. A future direction is to replace the SegNet backbone network with a more lightweight network to obtain speed improvements; another direction of research is to go for ways to enhance the effects of depth estimation.

## Figures and Tables

**Table 1:** Comparison on CityScapes dataset.

Architecture	#P	Segmentation		Depth	
		mIoU↑	Pix Acc↑	Abs Err↓	Rel Err↓
MTRAN	1.605 × 10^7^	**79.25**	**94.87**	**0.0218**	**38.0849**
Without-att	1.602 × 10^7^	79.22	94.81	0.0228	41.1473

**Table 2:** Comparison on NYUv2 dataset.

Architecture	#P	Segmentation		Depth	
		mIoU↑	Pix Acc↑	Abs Err↓	Rel Err↓
MTRAN	1.605 × 10^7^	**35.43**	**61.03**	**0.5278**	**0.2489**
Without-att	1.602 × 10^7^	35.14	60.25	0.5556	0.2675

**Table 3:** Results of our network structure and the MTAN on the NYUv2 dataset.

Architecture	#P	Segmentation		Depth	
		mIoU↑	Pix Acc↑	Abs Err↓	Rel Err↓
MTRAN	1.605 × 10^7^	**35.43**	**61.03**	**0.5278**	**0.2489**
MTAN [9]	4.121 × 10^7^	17.72	55.32	0.5906	0.2577

**Table 4:** Results on CityScapes dataset.

Architecture	#P	Segmentation		Depth	
		mIoU↑	Pix Acc↑	Abs Err↓	Rel Err↓
MTRAN	1.605 × 10^7^	**79.25**	**94.87**	**0.0218**	**38.0849**
Remove residuals	1.605 × 10^7^	79.14	94.83	0.0220	52.3582

**Table 5:** Comparison with and without optimization strategies.

Architecture	#P	Segmentation		Depth	
		mIoU↑	Pix Acc↑	Abs Err↓	Rel Err↓
MTRAN with IMTL-RWL	1.605 × 10^7^	79.25	94.87	**0.0218**	**38.0849**
MTRAN with IMTL	1.605 × 10^7^	**79.26**	**95.03**	0.0225	48.3591

**Table 6:** Results of different structures on the CityScapes dataset.

Architecture	#P	Segmentation		Depth	
		mIoU↑	Pix Acc↑	Abs Err↓	Rel Err↓
Single-Task	9.45 × 10^6^	77.94	94.38	0.0223	49.5168
MTAN [9]	4.121 × 10^7^	53.86	91.11	**0.0144**	33.63
Multi-Task	9.49 × 10^6^	78.03	94.48	0.0261	34.0978
DAMAN [34]	-	55.45	92.07	0.0146	**26.33**
Cross-Stitch [35]	2.163 × 10^7^	74.48	93.54	0.0257	47.9483
MTRAN	1.605 ×10^7^	**79.25**	**94.87**	0.0218	38.0849

**Table 7:** Performance of different optimization strategies on CityScapes dataset.

Architecture	#P	Segmentation		Depth	
		mIoU↑	Pix Acc↑	Abs Err↓	Rel Err↓
IMTL-RWL	1.605 × 10^7^	79.25	94.87	0.0218	**38.0849**
MGD [11]	1.605 × 10^7^	78.46	94.66	0.0220	53.3083
CAG [36]	1.605 × 10^7^	79.25	94.86	**0.0217**	48.4214
PCG [37]	1.605 × 10^7^	79.01	94.76	0.0220	55.1992
GradDrop [38]	1.605 × 10^7^	**79.47**	**94.92**	0.0220	48.7384

## Data Availability

The data presented in this study are available on request from the corresponding author.
